# Nighttime Systolic Blood Pressure and Cardiovascular and All-Cause Mortality in Peritoneal Dialysis Patients: A Single-Center Prospective Cohort Study

**DOI:** 10.3390/jcm15187300

**Published:** 2026-09-19

**Authors:** Jing Yu, Jianwen Yu, Rui Yang, Yagui Qiu, Jianbo Li, Xiao Yang, Wei Chen, Xi Xia, Qinghua Liu

**Affiliations:** 1 Department of Nephrology, The First Affiliated Hospital, Sun Yat-sen University, Guangzhou 510080, China; 2 Key Laboratory of Nephrology, Ministry of Health and Guangdong Province, Guangzhou 510080, China

**Keywords:** ambulatory blood pressure monitoring, nighttime systolic blood pressure, peritoneal dialysis, cardiovascular mortality, all-cause mortality

## Abstract

**Background/Objectives:** The link between ambulatory-blood-pressure-derived measurements and survival outcomes among patients receiving peritoneal dialysis (PD) remains uncertain. This study sought to explore whether nighttime systolic blood pressure (SBP) obtained from 24-h ambulatory blood pressure monitoring (ABPM) is associated with cardiovascular (CV) and all-cause mortality in PD individuals. **Methods:** Between 1 January 2017 and 30 June 2021, this single-center sub-cohort study (derived from a prospective database of PD) enrolled 165 PD participants with a median follow-up of 47.4 months from the date of ABPM completion. Competing risk regression was applied to estimate the association between nighttime SBP and CV mortality, accounting for competing death events. Multivariable Cox proportional hazards regression was utilized to quantify nighttime SBP’s independent correlation with all-cause mortality. **Results:** Over the follow-up period, 48 patients (29.1%) died, with 28 (58.3%) cases attributed to CV causes. In the fully adjusted model, each 1 SD rise in nighttime SBP corresponded to a subdistribution hazard ratio (SHR) of 1.53 (95% confidence interval [CI], 1.05–2.21; *p* = 0.026) for CV mortality. In the Cox regression model, every 1 SD increase in nighttime SBP showed an independent association with a 42% excess risk of all-cause mortality (hazard ratio [HR] = 1.42, 95% CI, 1.04–1.93; *p* = 0.026). Quartile-stratified analyses indicated that participants within the uppermost nighttime SBP quartile exhibited higher mortality risk for both endpoints compared to those in the lowest quartile. **Conclusions:** Elevated nighttime SBP is associated with increased CV and all-cause mortality risk in PD patients.

## 1. Introduction

Patients with end-stage renal disease (ESRD) face a disproportionate burden of cardiovascular (CV) mortality, which accounts for the majority of deaths in this population [1]. Hypertension affects 70–80% of these patients and represents the most prevalent modifiable CV risk factor, yet overall blood pressure (BP) control remains persistently suboptimal in this population [2].

Office BP has long served as the routine metric for clinical BP assessment and treatment titration, but it presents substantial limitations in dialysis populations. First, investigations relying on office or peridialytic BP measurements have repeatedly uncovered a paradoxical U-shaped mortality relationship, whereby low and high BP values are both associated with increased mortality risk [3,4]. This reverse epidemiology is largely driven by the underlying malnutrition, frailty, and severe comorbidities reflected by low office BP, rather than optimal BP control [3]. Second, single-occasion office readings cannot capture true 24-h BP and may miss prevalent abnormal BP phenotypes. Masked hypertension affects 19.3% to nearly 30% of PD patients [5,6]; although their target organ damage is comparable to that of sustained hypertension [7], their CV risk is routinely underestimated due to normal office BP values. Third, disrupted diurnal BP patterns—particularly a non-dipping status—are exceedingly common among PD patients and carry prognostic significance for adverse clinical events [8], which cannot be detected by office measurements alone.

Twenty-four-hour ambulatory blood pressure monitoring (ABPM) has emerged as the reference-standard modality for quantifying the genuine cumulative BP burden [9]. Data derived from both the general population and hemodialysis cohorts consistently demonstrate that nighttime BP exerts a more powerful prognostic influence on CV outcomes than either office or daytime BP measurements [9,10,11], a difference thought to stem from the sustained hemodynamic stress imposed on the heart and vasculature by uninterrupted nighttime BP elevation. In PD populations, a prior study observed that ABPM-derived 24-h average systolic BP (SBP) is independently associated with increased risks of CV and all-cause mortality [12]. Nevertheless, prior ABPM studies conducted in the PD population have predominantly examined 24-h average indices, and prospective data specifically examining the association between nighttime SBP and survival endpoints remain limited. Accordingly, the present investigation was designed to prospectively evaluate the associations between ABPM-assessed nighttime SBP and CV, as well as all-cause mortality, in a PD patient cohort.

## 2. Materials and Methods

### 2.1. Study Population

This study is a sub-cohort analysis of a large prospective PD cohort database maintained at the First Affiliated Hospital of Sun Yat-sen University. For this specific sub-study on ABPM, patient enrollment and data collection were performed between 1 January 2017 and 30 June 2021. During this enrollment period, a total of 565 incident PD patients were treated at our center. ABPM was not a routine clinical examination but was offered to eligible patients on a voluntary basis after detailed explanation of the study purpose and the patient provided written informed consent. A total of 187 patients met the basic eligibility criteria (age ≥ 18 years, PD duration ≥ 3 months) and agreed to undergo 24-h ABPM. Individuals were excluded if they had received a kidney transplant, had received hemodialysis for longer than three months, suffered from an active malignancy, or had a substantial amount of missing ABPM readings. The date of ABPM completion was defined as the index date (baseline) for each participant, and survival follow-up began from this index date to avoid immortal-time bias. After applying all eligibility filters, 165 participants were finally included for analysis, with follow-up data collected until 31 December 2025. All consecutive willing participants were recruited, with no additional selection based on BP levels, symptom severity, or other clinical characteristics. The study protocol followed the ethical principles laid out by the Declaration of Helsinki, and ethical clearance was granted by the Ethics Committee of the First Affiliated Hospital of Sun Yat-sen University.

### 2.2. Ambulatory BP Monitoring

Twenty-four-hour ABPM measurements were acquired using the TM-2430 upper-arm oscillometric device (A&D Company, Limited, Tokyo, Japan). All monitoring procedures were scheduled on working weekdays. Participants were permitted to carry out their ordinary daily routines but were advised to avoid heavy physical exertion. Participants were instructed to maintain their habitual sleep–wake rhythm throughout the whole monitoring window and remain stationary during each automated BP reading. The device was programmed to obtain readings at 20 min intervals during daytime hours and at 30 min intervals overnight. In alignment with contemporary ABPM guidelines [13], daytime was designated as 07:00–23:00 and nighttime as 23:00–07:00 using fixed clock-hour definitions. Individual sleep/wake diaries were not collected in this study. Valid ABPM tracings needed to contain ≥70% of the scheduled total measurements, and continuous gap readings could not exceed 3 h. BP target achievement was categorized using the following thresholds: 24 h average BP below 130/80 mmHg, daytime BP below 135/85 mmHg, and nighttime BP below 120/70 mmHg [13]. Study participants were not notified of their ABPM test results.

### 2.3. Office BP Measurement

Office-based BP readings were collected for every participant during inpatient or outpatient visits on working days, with no fasting requirement. Before measurement, participants rested quietly for 5–10 min. A clinically validated upper-arm oscillometric sphygmomanometer was used for each reading. The final office BP value for each participant was calculated as the average of two separate visits: one on the day the ABPM monitor was fitted and the other upon monitor retrieval. Office BP target achievement was defined as a reading below 140/90 mmHg [13]. All office BP assessments were performed by one dedicated PD-specialized nurse within our center.

### 2.4. Data Collection and Measurements

Baseline demographic characteristics, clinical information, and detailed medication histories were collected at the time of the initial hospital admission, together with comprehensive documentation of any pre-existing comorbid conditions. Diabetes mellitus was diagnosed according to established clinical criteria or confirmed in patients who were receiving ongoing anti-diabetic pharmacotherapy or insulin therapy. A history of prior CV events was ascertained through meticulous review of medical records, encompassing angina pectoris, myocardial infarction, heart failure, coronary revascularization procedures, and cerebrovascular events [14]. Hypertension status was assigned when subjects exhibited persistent BP elevations above 140/90 mmHg or were maintained on regular antihypertensive agents. In this PD cohort, hypertension was predominantly renal (secondary) hypertension; essential hypertension was uncommon and was not systematically distinguished.

Laboratory testing was conducted within the first three months following PD initiation. Test panels included hemoglobin, serum albumin (ALB), high-sensitivity C-reactive protein (Hs-CRP), serum creatinine, uric acid, and full lipid profiles. All biochemical assays were conducted following standardized laboratory operating procedures [14]. Serum creatinine-based estimated glomerular filtration rate (eGFR) was calculated using the Chronic Kidney Disease Epidemiology Collaboration (CKD-EPI) formula [15] for baseline characterization and secondary exploratory analyses. In addition, residual renal function (RRF) was measured from 24-h urine collections and calculated as the mean urea and creatinine clearance standardized to a 1.73 m^2^ body surface area.

### 2.5. Follow-Up

The principal exposure under investigation was nighttime systolic blood pressure (SBP), as determined by the ABPM recordings, which was evaluated both as a continuous parameter and as a categorical variable divided into quartiles. The primary study endpoint was CV mortality, with all-cause mortality designated as the secondary endpoint. CV mortality was operationally defined as any death attributable to cardiovascular or cerebrovascular pathology. The spectrum of relevant fatal etiologies included coronary heart disease (acute myocardial infarction, sudden cardiac death secondary to coronary atherosclerosis, ischemic heart failure, and fatal complications arising during invasive procedures for atherosclerotic disease), ischemic stroke, peripheral arterial disease, hemorrhagic stroke, nonischemic heart failure, primary arrhythmic disorders, valvular or pericardial cardiac disease, unexplained sudden death, and pulmonary embolism [16].

All mortality outcomes were collectively verified by attending physicians with formal board certification at our institution. The adjudication of each event relied on complete primary source materials, such as official death certificates, comprehensive inpatient charts, imaging findings (including coronary angiography, cranial CT/MRI, and vascular ultrasonography), and laboratory data supporting etiological diagnosis. In cases of out-of-hospital death lacking clinical workup records, investigators conducted telephone interviews with next-of-kin to clarify the circumstances surrounding the death. Study clinicians then assigned the cause of death after integrated evaluation of terminal manifestations, pre-existing medical conditions, recent health conditions, and information provided by family members [17,18].

Patient survival status was monitored by a specialized follow-up group composed of clinicians and professionally trained nurses. Monthly contact was maintained through telephone consultations or on-site interviews to track clinical status and adjust therapeutic plans as appropriate. Additionally, participants attended quarterly routine visits at our center for complete clinical assessments [18]. Person-time was calculated from the index date (ABPM completion) until the date of death, censoring, or 31 December 2025, whichever occurred first. Censoring definitions were specified as follows: participants who remained on continuous PD until the conclusion of the observation period were censored. Censoring also applied to individuals who shifted to hemodialysis, received kidney transplantation, transferred care to other medical facilities, or became lost to follow-up.

### 2.6. Statistical Analysis

Study participants were grouped according to baseline nighttime SBP quartile: quartile 1 (Q1): ≤125 mmHg; quartile 2 (Q2): >125 to ≤138 mmHg; quartile 3 (Q3): >138 to ≤151 mmHg; and quartile 4 (Q4): >151 mmHg. The lowest quartile (Q1) served as the reference group for all comparative analyses. The normality of continuous variables was formally assessed. Variables with a normal distribution are presented as the mean ± standard deviation (SD), while those exhibiting skewed distributions are reported as the median and interquartile range. Categorical variables are summarized using absolute frequencies and relative percentages. Between-group comparisons were performed using one-way analysis of variance for normally distributed continuous variables, the Kruskal–Wallis test for non-normally distributed continuous variables, and Pearson’s chi-square tests for categorical measures.

To identify factors potentially associated with elevated nighttime SBP, univariate logistic regression analyses were initially conducted as a screening step. Variables demonstrating a *p*-value below 0.20 in the univariate screening were subsequently entered into the multivariable logistic regression model using the Enter method [19]. The results derived from logistic regression modeling were expressed as odds ratios (ORs) and 95% confidence intervals (CIs).

For the purpose of illustrating the cumulative probability of CV mortality, cumulative incidence function (CIF) curves were generated, and between-group statistical comparisons were performed using the Fine–Gray test [20]. To evaluate the relationship between nighttime SBP and all-cause mortality, Kaplan–Meier survival curves were constructed, and inter-group differences were assessed using the log-rank test. Multivariable-adjusted restricted cubic spline regression with three knots placed at the 10th, 50th, and 90th percentiles of nighttime SBP (113, 138, and 162 mmHg, respectively) was used to visualize the continuous-scale relationships between nighttime SBP and both study endpoints. The spline curves were plotted over the 5th–95th percentile range of nighttime SBP (107–167 mmHg), with 125 mmHg (the upper boundary of Q1) serving as the reference value. A rug plot was added to display the distribution of individual observations.

To account for potential competing-event bias arising from the mutual exclusivity of CV-related and non-CV mortality, competing risk regression models were constructed to evaluate the relationship between nighttime SBP and CV mortality, with results expressed as subdistribution hazard ratios (SHRs) and 95% CIs. For all-cause mortality, Cox proportional hazards regression was employed to determine the independent association with nighttime SBP, reporting hazard ratios (HRs) and corresponding 95% CIs. Covariate selection was guided by established clinical significance and prior published evidence, and variables were introduced into three hierarchically adjusted models to evaluate the stability of the observed associations. Model 1 only included age and sex; Model 2 expanded upon Model 1 by additionally incorporating diabetes status, prior CV events, body mass index (BMI), and serum albumin; Model 3 included office SBP in addition to all Model 2 covariates. The proportional hazards assumption for the Cox regression models was assessed using Schoenfeld residual tests, and no significant violations were detected for any covariate (all *p* > 0.05). For the Fine–Gray competing risk regression models, the proportional subdistribution hazards assumption was similarly verified using Schoenfeld-type residuals, with no significant violations identified. Regarding missing data, complete-case analysis (i.e., listwise deletion) was applied to all analyses. None of the covariates entered into the multivariable models had missing values; accordingly, all 165 participants were included in each multivariable model. For descriptive analyses, variables with missing data were reported for those with available measurements. Statistical significance was defined as a two-sided *p*-value below 0.05. All data analyses were executed using SPSS version 26.0 and Stata version 14.0.

## 3. Results

### 3.1. Population

**Figure 1:**
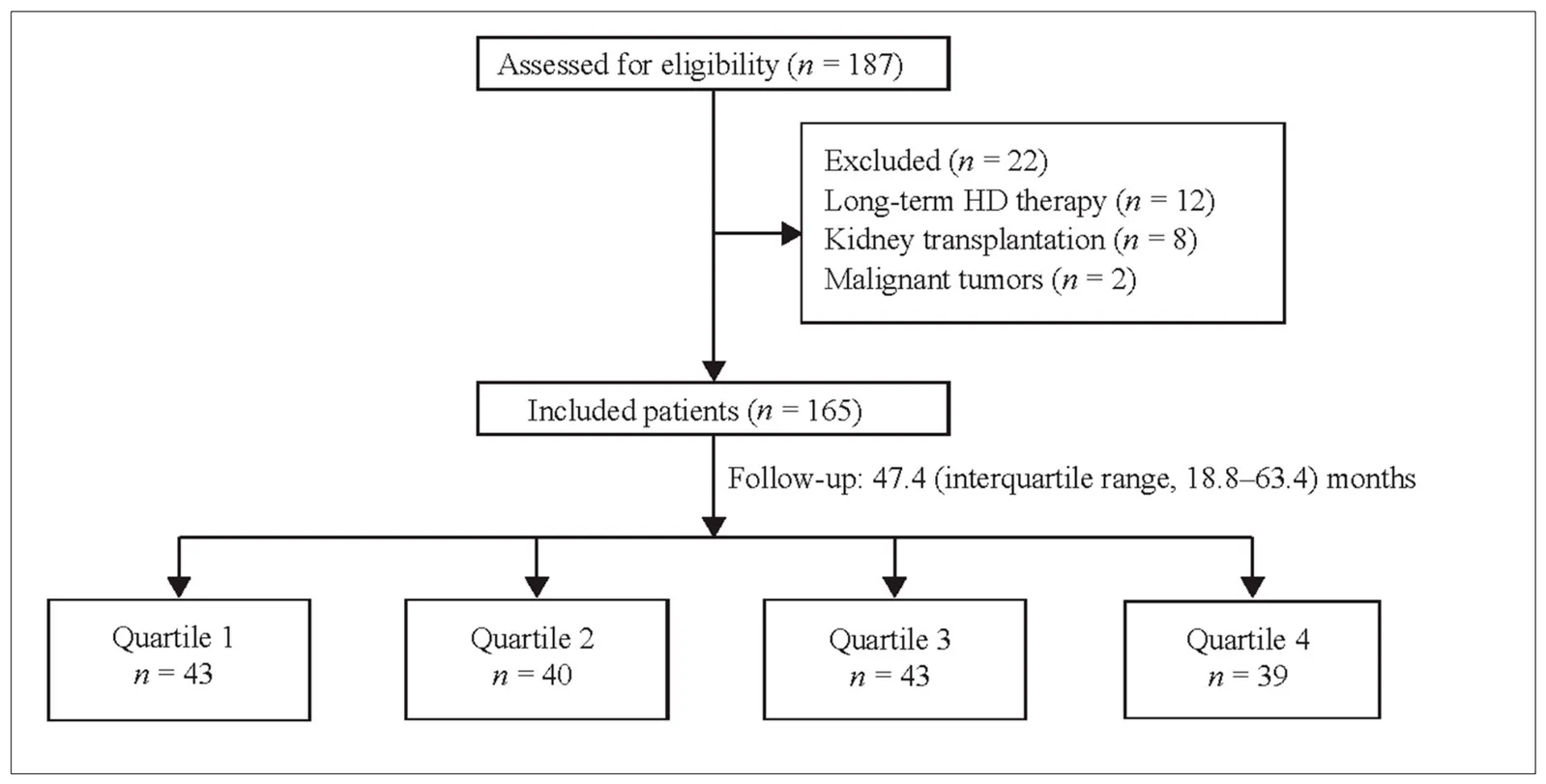
Patient selection flow chart. During the recruitment period, 565 incident PD patients were treated at our center. Of these, 187 eligible patients who agreed to undergo voluntary ABPM were initially assessed. After excluding 22 patients, 165 patients were included in the final analysis. Abbreviations: ABPM, ambulatory blood pressure monitoring; HD, hemodialysis; PD, peritoneal dialysis.

The final analytical cohort comprised 165 patients. Based on nighttime SBP measurements obtained from 24 h ABPM, participants were divided into four quartile groups, which contained 43, 40, 43, and 39 individuals, respectively. The complete patient selection process is illustrated in Figure 1, and the baseline clinical characteristics of the entire study population are detailed in Table 1. The overall cohort had a mean age of 42.4 ± 13.4 years, with males constituting 58.2% of participants and patients with diabetes representing 10.9% of the population. The mean nighttime SBP across the cohort was 137.8 ± 18.1 mmHg. Individuals assigned to the uppermost nighttime SBP quartile (Q4) demonstrated elevated concentrations of Hs-CRP, as well as higher readings for office SBP, office diastolic blood pressure (DBP), 24 h average SBP, 24 h average DBP, daytime SBP, daytime DBP, and nighttime DBP.

**Figure 2:**
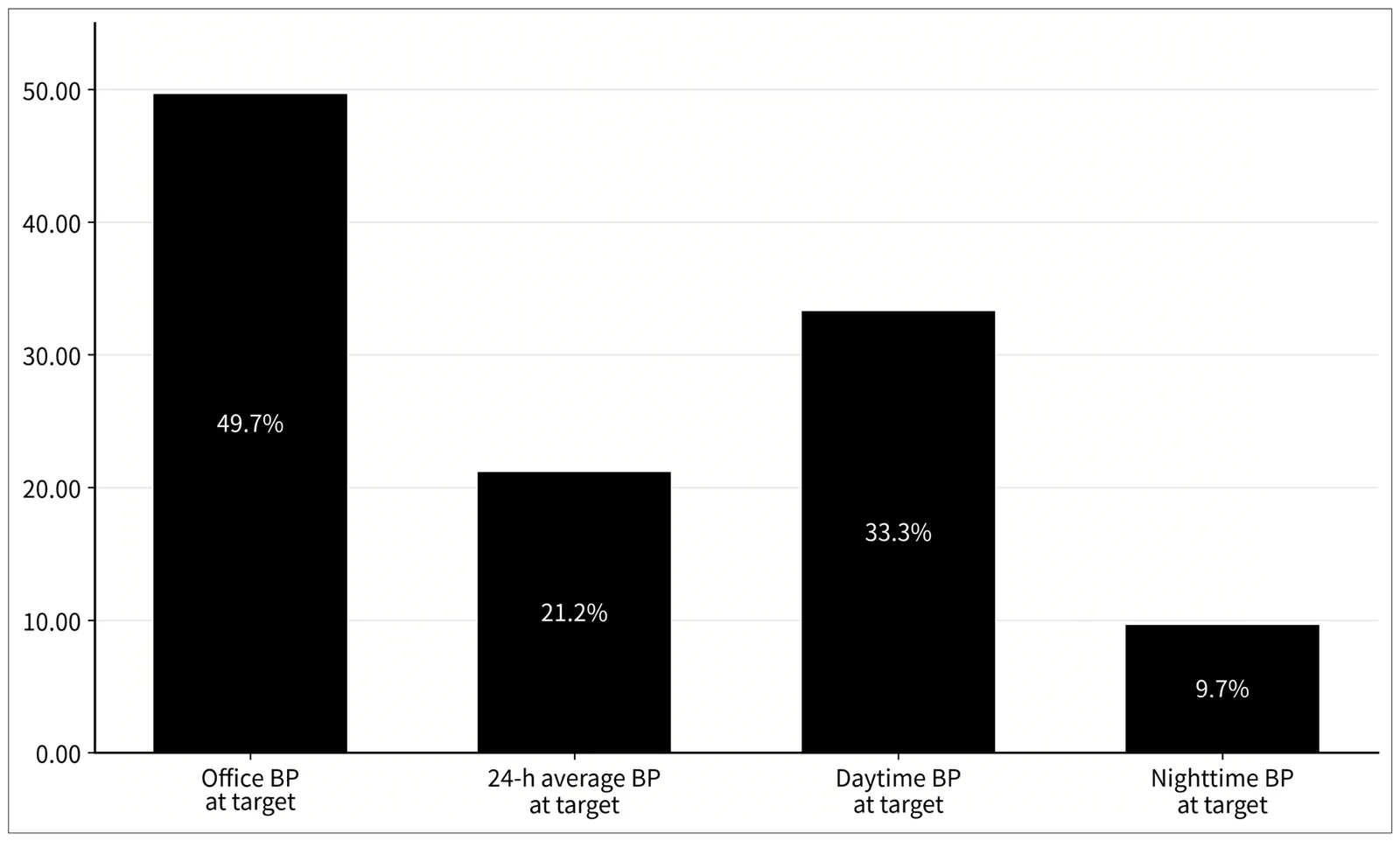
Percentages of patients achieving blood pressure targets according to various blood-pressure measurement criteria. Abbreviations: BP, blood pressure.

Over a median follow-up period of 47.4 months, 48 participants (29.1%) died, among whom cardiovascular-related fatalities constituted 58.3% of all deaths. All-cause mortality rates across Q1–Q4 were 9.3%, 15.0%, 27.9%, and 66.7%, respectively; the corresponding CV mortality rates were 9.3%, 7.5%, 11.6%, and 41.0%, respectively. Overall BP control was suboptimal within this cohort. Slightly under half (49.7%) of the participants satisfied the predefined office BP target. By comparison, target compliance derived from ABPM metrics was markedly poorer: 21.2% for 24 h average BP, 33.3% for daytime BP, and merely 9.7% for nighttime BP (Figure 2). Cross-classification of office and nighttime BP control status revealed that 41.8% of the cohort had isolated nocturnal hypertension (controlled office BP but uncontrolled nighttime BP), 48.5% had uncontrolled office and nighttime BP, 7.9% achieved both targets, and only 1.8% exhibited a white-coat effect (uncontrolled office BP but controlled nighttime BP).

### 3.2. Factors Associated with Elevated Nighttime SBP

Table 2 presents the factors associated with higher nighttime SBP levels (quartile 4). In univariate logistic regression, older age was significantly associated with higher nighttime SBP (OR: 1.03; 95% CI, 1.00–1.06; *p* = 0.030), whereas higher BMI (OR: 1.10; 95% CI, 0.98–1.23; *p* = 0.118) and lower eGFR (OR: 0.87; 95% CI, 0.72–1.05; *p* = 0.138) showed nonsignificant trends toward association. Consistent with our prespecified variable selection strategy, covariates with *p* < 0.20 in the univariate analysis, namely, age, BMI, and eGFR, were inputted into the multivariable logistic regression model using the Enter method. After multivariable adjustment, advancing age showed a trend toward association with elevated nighttime SBP (adjusted OR: 1.03; 95% CI, 1.00–1.05; *p* = 0.090), while BMI (adjusted OR: 1.06; 95% CI, 0.94–1.20; *p* = 0.317) and eGFR (adjusted OR: 0.90; 95% CI, 0.74–1.10; *p* = 0.305) were not significantly associated with nighttime SBP in the multivariable model. No independent correlate of elevated nighttime SBP was identified at the conventional *p* < 0.05 threshold.

### 3.3. Association Between Nighttime SBP and CV and All-Cause Mortality

**Figure 3:**
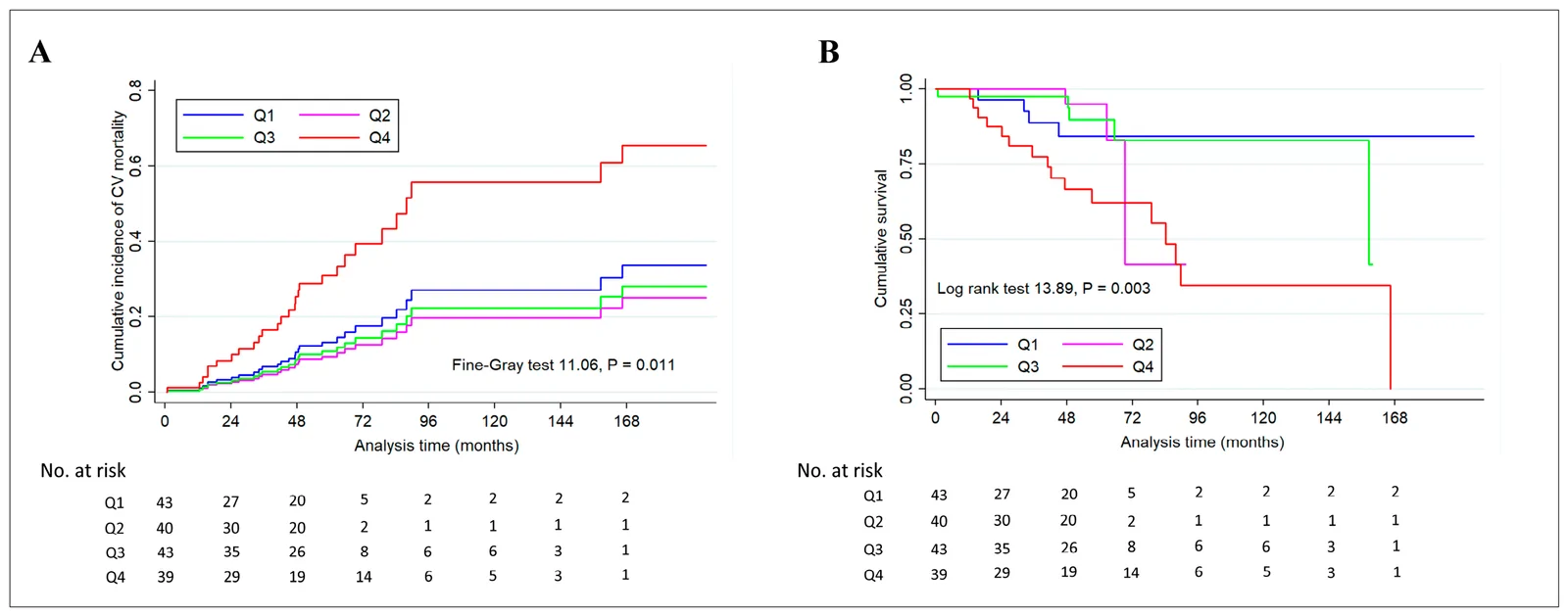
Cumulative incidence function and Kaplan–Meier survival curve analyses. Panel (**A**) illustrates the cumulative incidence of CV mortality stratified by nighttime SBP quartile. Panel (**B**) depicts the survival probability for all-cause mortality across nighttime SBP quartiles. Abbreviations: CV, cardiovascular; Q1–Q4, lowest to highest quartile; SBP, systolic blood pressure.

Analysis of the CIF curves indicated that participants in the uppermost quartile had a significantly greater cumulative risk of CV mortality compared to individuals in the remaining three quartiles (*p* = 0.011, Figure 3A). Correspondingly, Kaplan–Meier survival curve analysis demonstrated that the highest quartile was associated with the greatest risk of all-cause mortality (*p* = 0.003, Figure 3B).

**Figure 4:**
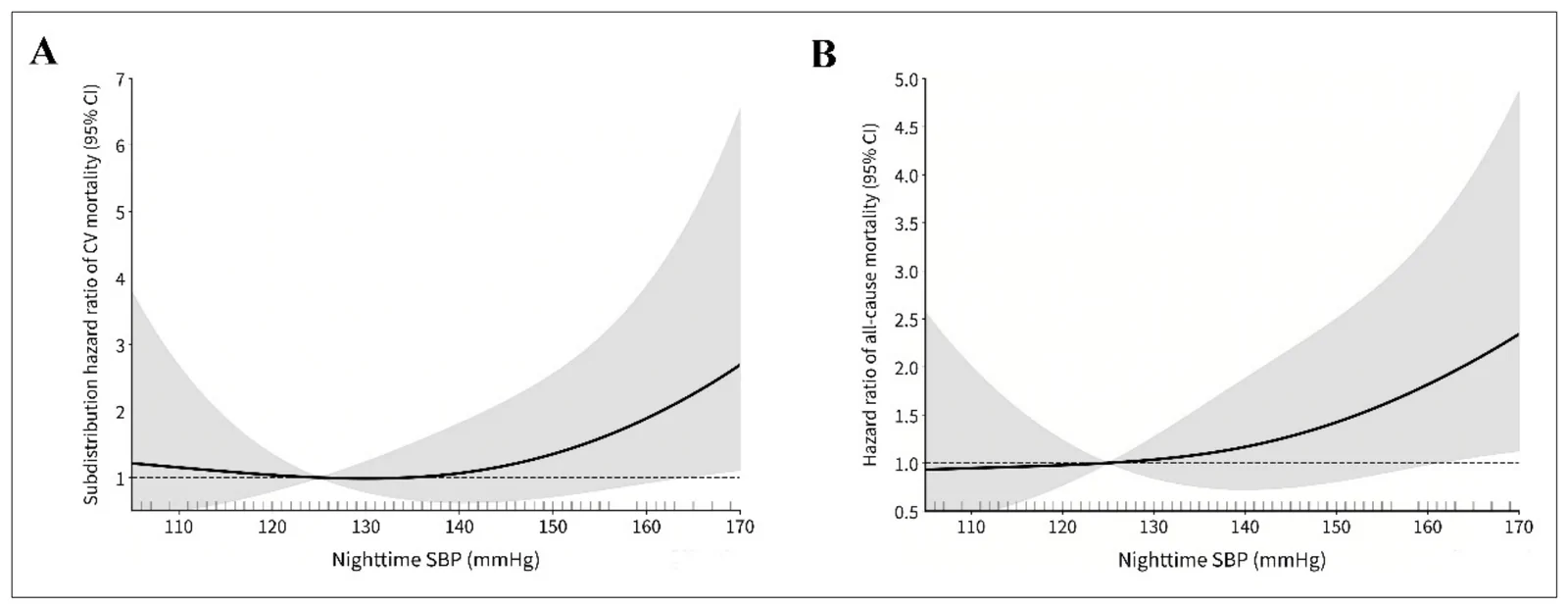
Restricted cubic splines with three knots. Association of nighttime SBP with multivariable-adjusted CV mortality (**A**) and all-cause mortality (**B**). The solid black line represents the fitted spline estimate of the subdistribution hazard ratio (SHR) or hazard ratio (HR), and the dashed horizontal line indicates the null reference line (SHR = 1 or HR = 1). Knots were placed at the 10th, 50th, and 90th percentiles of nighttime SBP (113, 138, and 162 mmHg). The x-axis displays the 5th–95th percentile range (107–167 mmHg), with 125 mmHg as the reference. The rug plot at the bottom indicates the distribution of individual nighttime SBP values. Shaded areas represent 95% confidence intervals. Abbreviations: CI, confidence interval; CV, cardiovascular; SBP, systolic blood pressure.

Restricted cubic spline analysis with three knots revealed a tendency toward a J-shaped pattern between nighttime SBP and CV mortality (Figure 4A), with a slight, non-significant elevation in risk at lower SBP levels (<125 mmHg), a nadir around 130–140 mmHg, and a steep increase above 145 mmHg. However, this J-shaped pattern was not statistically significant (*p* for nonlinearity = 0.250), and the confidence intervals were particularly wide at the lower end of the nighttime SBP range, indicating substantial uncertainty in this region. Regarding all-cause mortality (Figure 4B), the association was approximately monotonic and positive, with no significant deviation from linearity (*p* for nonlinearity = 0.524); the risk curve was relatively flat below 140 mmHg and rose progressively thereafter.

The relationships between nighttime SBP and both CV and all-cause mortality are presented in Table 3. In the fully adjusted model (Model 3), competing risk regression demonstrated that each 1 SD increase in nighttime SBP was associated with a 53% increase in CV mortality risk (SHR: 1.53; 95% CI, 1.05–2.21; *p* = 0.026). Of note, this association did not reach statistical significance in the less-adjusted models (Model 1: *p* = 0.297; Model 2: *p* = 0.161). In the quartile-stratified analyses using quartile 1 as the reference category, the adjusted SHR estimates were 0.81 (95% CI, 0.15–4.32; *p* = 0.810) for quartile 2, 0.97 (95% CI, 0.23–4.09; *p* = 0.970) for quartile 3, and 3.81 (95% CI, 1.16–12.57; *p* = 0.028) for quartile 4. Regarding all-cause mortality, multivariable Cox proportional hazards regression revealed that each 1 SD increase in nighttime SBP was associated with a 42% increase in all-cause mortality risk (HR: 1.42; 95% CI, 1.04–1.93; *p* = 0.026). In the comparisons with quartile 1, the adjusted HRs were 1.58 (95% CI, 0.43–5.73; *p* = 0.488) for quartile 2, 1.70 (95% CI, 0.51–5.63; *p* = 0.387) for quartile 3, and 4.16 (95% CI, 1.33–12.95; *p* = 0.014) for quartile 4.

## 4. Discussion

Our study found that elevated nighttime SBP was associated with increased CV and all-cause mortality. In the fully adjusted model, the association with CV mortality reached statistical significance after additional adjustment for office SBP, although it did not reach significance in the less-adjusted models. Notably, the use of Fine–Gray competing risk regression in our analysis accounted for the competing effect of non-CV mortality, which lends greater robustness to the estimates of CV mortality risk.

Our findings broadly align with the existing evidence regarding ABPM in dialysis populations. Accumulating data indicate that interdialytic ABPM offers greater prognostic information than peridialytic BP recordings [21,22]. While dialysis-unit BP measurements consistently display a U-shaped association with mortality [3,4], some studies using standardized ABPM have reported a predominantly positive or linear association between ambulatory BP and mortality in dialysis patients, similar to that observed in the general population [23]. One study on a PD-specific cohort reported that 24 h SBP was independently associated with CV mortality and CV events [12]. In our restricted cubic spline analyses, all-cause mortality showed a monotonic positive association with nighttime SBP without significant deviation (*p* for nonlinearity = 0.524), whereas CV mortality exhibited a non-significant tendency toward a J-shaped pattern (*p* for nonlinearity = 0.250), with a slight elevation in risk at lower SBP levels and a steep increase above 145 mmHg. Our study extends this body of evidence and confirms that nighttime SBP constitutes a risk factor for adverse outcomes in PD patients.

Nevertheless, several earlier studies using office or peridialytic BP have reported a U-shaped association between BP and mortality in dialysis patients [3,4]. In our cohort, a clear U-shaped pattern was not observed for all-cause mortality; however, a non-significant J-shaped trend was noted for CV mortality, with a slight, non-significant elevation in risk at lower nighttime SBP levels (<125 mmHg). This pattern may be attributed to three factors. First, the slight, statistically nonsignificant elevation in CV mortality at lower SBP levels could potentially be explained in part by reverse causality: low BP in dialysis patients may be a marker of underlying frailty, malnutrition, or advanced cardiac dysfunction rather than a causal risk factor [3,4]. However, this J-shaped pattern was not statistically supported (*p* for nonlinearity = 0.250), and the confidence intervals were wide at the lower end of nighttime SBP, indicating considerable uncertainty. Reverse causality therefore remains a speculative hypothesis rather than a definitive explanation for this observed visual trend. Our cohort was relatively young, with a low prevalence of diabetes and advanced CV comorbidities; thus, confounding from frailty, malnutrition, and severe comorbidities, which drive excess mortality at low BP levels, was less prominent, consistent with the observations of Georgianos et al. [12]. Second, the BP–mortality relationship is time-dependent: low BP is more closely linked to early mortality, whereas the detrimental effects of elevated BP emerge and predominate with longer follow-up [24]. With a median follow-up of nearly 4 years, the long-term adverse effects of sustained hypertension on CV structure are more readily detectable in our cohort, in line with prior PD cohort findings showing that high BP confers increased late mortality [24]. Third, nighttime SBP measured via standardized ABPM is less susceptible to measurement bias than office or peridialytic readings, and thus more accurately reflects true long-term BP load [21,22].

Several pathophysiological pathways may underlie the association between elevated nighttime SBP and adverse outcomes. First, subclinical volume overload is a central driver of hypertension in PD patients and a key contributor to elevated nighttime SBP. Previous bioimpedance studies have documented subclinical hypervolemia in more than 30% of PD patients [25]. In the supine position during sleep, redistribution of extracellular fluid increases venous return and cardiac output, raising nighttime SBP and blunting the normal nocturnal dipping pattern. Chronic volume overload persistently elevates left ventricular afterload, promoting left ventricular hypertrophy, diastolic dysfunction, and ultimately heart failure and CV mortality [26]. Second, autonomic dysfunction and sympathetic overactivity are highly prevalent in ESRD [27]. Abnormal nocturnal BP rhythms are a direct manifestation of impaired autonomic regulation, and sustained sympathetic activation contributes to adverse myocardial remodeling, arrhythmias, and sudden cardiac death—major contributors to CV mortality in dialysis populations [28]. Third, uninterrupted BP elevation throughout the night exposes the heart and vasculature to continuous hemodynamic stress, accelerating arterial stiffening and vascular calcification, which are core pathological substrates of CV events in PD patients [29,30]. In our cohort, patients in the highest nighttime SBP quartile had higher Hs-CRP levels, suggesting that chronic inflammation may also participate in this vicious cycle between nighttime SBP elevation and end-organ damage [31].

Clinically, nearly half of the patients in our cohort achieved the office BP target, whereas fewer than 10% met the nighttime BP target. Cross-classification further demonstrated that 41.8% of the patients had isolated nocturnal hypertension, indicating that a substantial proportion of patients with elevated nighttime BP levels are missed by routine office assessment alone. Our findings therefore support wider implementation of ABPM in routine PD clinical practice to accurately characterize true BP control and identify high-risk individuals. For patients with elevated nighttime SBP, priority should be given to volume optimization strategies, including adjustment of PD regimens, dietary sodium restriction, and careful dry weight titration [31]. Chronotherapeutic approaches, such as bedtime administration of long-acting antihypertensive agents, have been proposed as a potential strategy to improve nighttime BP control; however, the current evidence is insufficient to support routine bedtime dosing for improving CV outcomes, and this approach remains investigational, particularly in PD patients in whom dedicated trials are lacking [32,33].

Several limitations of this study should be acknowledged. First, this was a single-center prospective observational study with a relatively modest sample size. The fully adjusted competing-risk model for CV mortality only included 28 CV events and seven covariates, which limits the precision of effect estimates and increases the potential for model instability. Of note, the association between nighttime SBP and CV mortality reached statistical significance only in the fully adjusted Model 3 (*p* = 0.026), but not in Model 1 (*p* = 0.297) or Model 2 (*p* = 0.161); this inconsistency across adjusted models further indicates that the CV-mortality-related findings should be cautiously interpreted. Additionally, the voluntary nature of ABPM participation (33% participation rate) may introduce selection bias and limit generalizability, although recruitment was consecutive among willing participants and the cohort’s baseline characteristics were comparable to other reported PD cohorts. Second, ABPM was performed only at baseline, without longitudinal follow-up data on BP trajectories. Third, we did not adjust for volume status or the dosage and timing of antihypertensive medications—key determinants of nighttime BP in PD patients—which may have introduced residual confounding. However, a sensitivity analysis further adjusting for the number of antihypertensive drug classes yielded consistent results, suggesting the robustness of the results to treatment intensity. Additionally, unmeasured confounders—including nocturnal alcohol intake, poor sleep quality, untreated sleep apnea, high salt intake, psychosocial stress, and medication non-adherence—were not available in our database and therefore could not be adjusted for in the multivariable models, and the observed association should be interpreted as an epidemiological association rather than a causal relationship. Fourth, the relatively low comorbidity burden warrants caution when extrapolating to older, more comorbid, or prevalent PD populations. Fifth, methodologic limitations include potential survivor bias from the three-month minimum PD duration requirement (mitigated by initiating follow-up at the ABPM completion date to avoid immortal-time bias), and possible exposure misclassification from using fixed clock hours (23:00–07:00) rather than individual sleep periods to define nighttime BP, although this is the guideline-recommended standard approach.

## 5. Conclusions

In conclusion, this study found that elevated nighttime SBP is associated with increased risk of CV and all-cause mortality in PD patients, and this association persists after adjustment for office SBP. Future multicenter prospective cohorts are needed to validate these findings, and intervention trials targeting nighttime SBP control are warranted to determine whether optimizing nighttime BP can translate into improved long-term survival in PD patients.

## Figures and Tables

**Table 1:** Baseline characteristics of study cohort.

Covariate		Quartiles of Nighttime SBP (mmHg)				*p* Value
	Total (*n* = 165)	Q1 (≤125) (*n* = 43)	Q2 (>125 to ≤138) (*n* = 40)	Q3 (>138 to ≤151) (*n* = 43)	Q4 (>151) (*n* = 39)	
Age (years)	42.4 ± 13.4	40.3 ± 12.9	39.5 ± 13.5	43.5 ± 13.1	46.5 ± 13.5	0.073
Male (*n* (%))	96 (58.2)	27 (62.8)	24 (60.0)	22 (51.2)	23 (59.0)	0.727
Smoking (*n* (%))	44 (26.7)	10 (23.3)	7 (17.5)	14 (32.6)	13 (33.3)	0.305
Diabetes (*n* (%))	18 (10.9)	4 (9.3)	3 (7.5)	5 (11.6)	6 (15.4)	0.674
Prior CV events (*n* (%))	16 (9.7)	5 (11.6)	4 (10.0)	3 (7.0)	4 (10.3)	0.904
Hypertension (*n* (%))	156 (94.5)	39 (90.7)	38 (95.0)	41 (95.3)	38 (97.4)	0.586
BMI (kg/m^2^)	21.9 ± 3.1	21.4 ± 3.4	22.1 ± 3.4	21.4 ± 2.8	22.5 ± 2.9	0.285
Hemoglobin (g/L)	90.0 ± 20.0	92.9 ± 21.8	93.3 ± 21.5	85.0 ± 16.2	87.1 ± 19.3	0.138
Serum albumin (g/L)	32.9 ± 4.7	34.9 ± 4.6	31.7 ± 4.7	32.2 ± 4.0	32.5 ± 4.8	0.008
Hs-CRP (mg/L)	2.25 [0.78–7.05]	1.50 [0.72–3.37]	2.93 [0.92–7.09]	1.96 [0.53–12.62]	3.44 [1.26–10.02]	0.058
TC (mg/dL)	172.8 ± 51.5	178.7 ± 41.8	170.1 ± 64.0	172.7 ± 51.3	169.3 ± 48.6	0.839
TG (mg/dL)	121.2 [92.0–169.0]	126.6 [98.2–167.3]	124.8 [97.6–178.3]	114.2 [81.4–156.6]	115.9 [87.6–163.7]	0.246
HDL-C (mg/dL)	43.0 ± 13.4	45.4 ± 12.6	41.8 ± 16.6	41.7 ± 13.3	43.2 ± 10.6	0.554
LDL-C (mg/dL)	105.3 ± 35.8	107.7 ± 32.0	100.5 ± 41.1	109.9 ± 35.5	102.6 ± 35.0	0.603
Uric acid (mmol/L)	437.3 ± 119.5	450.8 ± 109.4	403.2 ± 110.4	459.9 ± 134.4	432.3 ± 117.8	0.143
eGFR (mL/min/1.73 m^2^)	5.5 ± 2.1	6.2 ± 1.9	5.6 ± 2.3	5.2 ± 1.6	5.1 ± 2.3	0.044
RRF (mL/min/1.73 m^2^)	3.89 [2.08–5.70]	4.40 [2.86–5.84]	3.91 [2.17–5.82]	3.30 [1.93–5.20]	3.35 [1.90–8.65]	0.504
Office SBP (mmHg)	138.8 ± 20.6	125.3 ± 14.9	133.8 ± 16.3	144.0 ± 17.9	153.2 ± 22.0	<0.001
Office DBP (mmHg)	86.0 ± 14.1	78.0 ± 11.9	82.5 ± 9.0	89.2 ± 14.0	94.9 ± 14.8	<0.001
24 h average SBP (mmHg)	139.7 ± 16.4	121.1 ± 8.4	133.9 ± 5.8	145.6 ± 7.6	159.6 ± 10.9	<0.001
24 h average DBP (mmHg)	84.2 ± 10.3	74.3 ± 8.0	81.6 ± 5.3	87.2 ± 8.6	94.2 ± 6.4	<0.001
Daytime SBP (mmHg)	140.5 ± 16.8	123.6 ± 10.0	134.8 ± 7.8	146.2 ± 10.5	158.8 ± 13.5	<0.001
Daytime DBP (mmHg)	84.9 ± 10.4	76.3 ± 9.0	82.2 ± 6.0	87.7 ± 9.4	94.2 ± 7.3	<0.001
Nighttime SBP (mmHg)	137.8 ± 18.1	115.4 ± 7.0	131.8 ± 3.8	144.2 ± 3.9	161.6 ± 9.6	<0.001
Nighttime DBP (mmHg)	82.5 ± 11.5	70.1 ± 6.7	80.2 ± 5.8	86.1 ± 8.0	94.4 ± 8.5	<0.001
Antihypertensive drugs (*n* (%))	153 (92.7)	38 (88.4)	38 (95.0)	40 (93.0)	37 (94.9)	0.618
Statin use (*n* (%))	34 (20.6)	11 (25.6)	6 (15.0)	7 (16.3)	10 (25.6)	0.473

Notes: Normally distributed data are expressed as the mean ± standard deviation (SD), whereas non-normal data are presented as the median and interquartile percentile ranging from 25% to 75%. The distribution of categorical variables is displayed as absolute frequencies and relative percentages. Abbreviations: BMI, body mass index; CV, cardiovascular; DBP, diastolic blood pressure; eGFR, estimated glomerular filtration rate; HDL-C, high-density lipoprotein cholesterol; Hs-CRP, hypersensitive C-reactive protein; LDL-C, low-density lipoprotein cholesterol; Q1–Q4, lowest to highest quartile; RRF, residual renal function; SBP, systolic blood pressure; TC, total cholesterol; TG, triglycerides.

**Table 2:** Factors associated with elevated nighttime SBP from univariate and multivariable analyses.

Variable	Univariate Logistic Regression		Multivariate Logistic Regression	
	Crude OR (95% CI)	*p* Value	Adjusted OR (95% CI)	*p* Value
Age (per 1-year increase)	1.03 (1.00–1.06)	0.030	1.03 (1.00–1.05)	0.090
Sex (female/male)	0.96 (0.46–1.99)	0.909		
Smoking (yes/no)	1.53 (0.70–3.34)	0.283		
Diabetes (yes/no)	1.78 (0.62–5.12)	0.284		
Prior CV events (yes/no)	1.09 (0.33–3.58)	0.893		
BMI (per 1 kg/m^2^ increase)	1.10 (0.98–1.23)	0.118	1.06 (0.94–1.20)	0.317
Hemoglobin (per 1 g/L increase)	0.99 (0.97–1.01)	0.375		
Serum albumin (per 1 g/L increase)	0.98 (0.91–1.06)	0.609		
Ln (Hs-CRP) (per 1-unit increase)	1.17 (0.90–1.52)	0.231		
eGFR (per 1 mL/min/1.73 m^2^ increase)	0.87 (0.72–1.05)	0.138	0.90 (0.74–1.10)	0.305
Ln (RRF) (per 1-unit increase)	0.91 (0.55–1.50)	0.715		

Notes: Higher nighttime SBP levels refer to quartile 4. Hs-CRP and RRF were natural log-transformed prior to regression analysis. Variables with *p* < 0.20 in univariate analysis (age, BMI, and eGFR) were included in the multivariable logistic regression model using the Enter method. All variables entered into the multivariable model are reported with their adjusted ORs, 95% CIs, and *p* values. The effective sample size for the multivariable logistic regression model was 165, as none of the covariates entered into the model had missing values. None of the variables reached conventional statistical significance in the multivariable model, although age showed a trend toward an association. Abbreviations: BMI, body mass index; CI, confidence interval; CV, cardiovascular; eGFR, estimated glomerular filtration rate; Hs-CRP, hypersensitive C-reactive protein; OR, odds ratio; RRF, residual renal function; SBP, systolic blood pressure.

**Table 3:** Association of nighttime SBP with CV and all-cause mortality.

	Per 1 SD Increase		Q2 (*n* = 40)		Q3 (*n* = 43)		Q4 (*n* = 39)	
	SHR (95% CI)	*p* Value	SHR (95% CI)	*p* Value	SHR (95% CI)	*p* Value	SHR (95% CI)	*p* Value
CV mortality								
Unadjusted	1.37 (0.91–2.08)	0.133	0.70 (0.16–3.11)	0.639	0.81 (0.22–3.00)	0.748	2.59 (0.83–8.08)	0.101
Model 1	1.25 (0.82–1.92)	0.297	0.68 (0.15–3.18)	0.623	0.52 (0.13–2.09)	0.359	2.18 (0.71–6.73)	0.176
Model 2	1.35 (0.89–2.04)	0.161	0.71 (0.14–3.56)	0.680	0.68 (0.17–2.70)	0.583	2.25 (0.73–6.96)	0.159
Model 3	1.53 (1.05–2.21)	0.026	0.81 (0.15–4.32)	0.810	0.97 (0.23–4.09)	0.970	3.81 (1.16–12.57)	0.028
	**HR (95% CI)**	***p*** **value**	**HR (95% CI)**	***p*** **value**	**HR (95% CI)**	***p*** **value**	**HR (95% CI)**	***p*** **value**
All-cause mortality								
Unadjusted	1.40 (1.09–1.82)	0.010	1.82 (0.51–6.51)	0.358	2.04 (0.65–6.38)	0.221	4.68 (1.61–13.56)	0.005
Model 1	1.36 (1.04–1.78)	0.024	1.78 (0.50–6.34)	0.376	1.63 (0.51–5.21)	0.408	4.14 (1.43–12.04)	0.009
Model 2	1.43 (1.07–1.91)	0.017	1.54 (0.43–5.54)	0.510	1.63 (0.50–5.26)	0.417	3.89 (1.32–11.45)	0.014
Model 3	1.42 (1.04–1.93)	0.026	1.58 (0.43–5.73)	0.488	1.70 (0.51–5.63)	0.387	4.16 (1.33–12.95)	0.014

Notes: The lowest quartile (Q1) was the reference group (*n* = 43). All multivariable models (Model 1, Model 2, and Model 3) included 165 participants, as none of the covariates entered into these models had missing values; missing data were handled using complete-case analysis (listwise deletion). Model 1: Adjusted for age and sex (*n* = 165). Model 2: Same as Model 1 plus adjustment for diabetes, prior CV events, body mass index, and serum albumin (*n* = 165). Model 3: Same as Model 2 plus adjustment for office SBP (*n* = 165). Abbreviations: CI, confidence interval; CV, cardiovascular; HR, hazard ratio; Q1 to Q4, lowest to highest quartile; SBP, systolic blood pressure; SD, standard deviation; SHR, subdistribution hazard ratio.

## Data Availability

The data presented in this study are available on request from the corresponding author. The data are not publicly available due to patient privacy and ethical restrictions.

## References

[1] Shoji T., Wanner C. (2026). Two-Step Clinical Pathways to Cardiovascular Mortality in Chronic Kidney Disease and Dialysis—A Narrative Review. J. Atheroscler. Thromb..

[2] Bucharles S.G.E., Wallbach K.K.S., Moraes T.P., Pecoits-Filho R. (2019). Hypertension in patients on dialysis: Diagnosis, mechanisms, and management. J. Bras. Nefrol..

[3] Georgianos P.I., Agarwal R. (2017). Blood Pressure and Mortality in Long-Term Hemodialysis-Time to Move Forward. Am. J. Hypertens..

[4] Zager P.G., Nikolic J., Brown R.H., Campbell M.A., Hunt W.C., Peterson D., Van Stone J., Levey A., Meyer K.B., Klag M.J. (1998). “U” curve association of blood pressure and mortality in hemodialysis patients. Kidney Int..

[5] Vareta G., Georgianos P.I., Vaios V., Sgouropoulou V., Roumeliotis S., Georgoulidou A., Dounousi E., Eleftheriadis T., Papagianni A., Balaskas E.V. (2022). Epidemiology of Hypertension among Patients on Peritoneal Dialysis Using Standardized Office and Ambulatory Blood Pressure Recordings. Am. J. Nephrol..

[6] Drawz P.E., Alper A.B., Anderson A.H., Brecklin C.S., Charleston J., Chen J., Deo R., Fischer M.J., He J., Hsu C.Y. (2016). Masked Hypertension and Elevated Nighttime Blood Pressure in CKD: Prevalence and Association with Target Organ Damage. Clin. J. Am. Soc. Nephrol..

[7] Caliskan M., Ciftci O., Gullu H., Caliskan Z., Güven A., Erdogan D., Muderrisoglu H. (2013). Effect of masked, white-coat, and sustained hypertension on coronary flow reserve and peripheral endothelial functions. Clin. Exp. Hypertens..

[8] Cader R.A., Gafor H.A., Mohd R., Ibrahim S., Wan Haslina W.H., Bain A., Kong N.C. (2012). Blood pressure profile in continuous ambulatory peritoneal dialysis patients. EXCLI J..

[9] Parati G., Pengo M.F., Avolio A., Azizi M., Bothe T.L., Burnier M., Cappuccio F.P., Sierra A., Fava C., Gironacci M.M. (2025). Nocturnal blood pressure: Pathophysiology, measurement and clinical implications. Position paper of the European Society of Hypertension. J. Hypertens..

[10] Hermida R.C., Crespo J.J., Otero A., Domínguez-Sardiña M., Moyá A., Ríos M.T., Castiñeira M.C., Callejas P.A., Pousa L., Sineiro E. (2018). Asleep blood pressure: Significant prognostic marker of vascular risk and therapeutic target for prevention. Eur. Heart J..

[11] Amar J., Vernier I., Rossignol E., Bongard V., Arnaud C., Conte J.J., Salvador M., Chamontin B. (2000). Nocturnal blood pressure and 24-hour pulse pressure are potent indicators of mortality in hemodialysis patients. Kidney Int..

[12] Georgianos P.I., Vaios V., Zebekakis P.E., Liakopoulos V. (2021). The Relation of Clinic and Ambulatory BP with the Risk of Cardiovascular Events and All-Cause Mortality among Patients on Peritoneal Dialysis. J. Clin. Med..

[13] Williams B., Mancia G., Spiering W., Rosei E.A., Azizi M., Burnier M., Clement D.L., Coca A., de Simone G., Dominiczak A. (2019). 2018 ESC/ESH Guidelines for the management of arterial hypertension. Kardiol. Pol..

[14] Yu J., Xia X., Huang N.Y., Qiu Y.G., Yang X., Mao H.P., Chen W., Huang F.X. (2022). Association of Ratio of Apolipoprotein B to Apolipoprotein A1 With Survival in Peritoneal Dialysis. Front. Nutr..

[15] Levey A.S., Stevens L.A., Schmid C.H., Zhang Y.L., Castro A.F., Feldman H.I., Kusek J.W., Eggers P., Van Lente F., Greene T. (2009). A new equation to estimate glomerular filtration rate. Ann. Intern. Med..

[16] Virani S.S., Alonso A., Aparicio H.J., Benjamin E.J., Bittencourt M.S., Callaway C.W., Carson A.P., Chamberlain A.M., Cheng S., Delling F.N. (2021). Heart Disease and Stroke Statistics-2021 Update: A Report From the American Heart Association. Circulation.

[17] Yu J., Xia X., Lin T., Huang N., Qiu Y., Yang X., Mao H., Chen W., Huang F. (2021). Non-high-density lipoprotein cholesterol and mortality among peritoneal dialysis patients. J. Clin. Lipidol..

[18] Zhang Y., Liang J., Li S., Ma K., Zhang D., Chen W., Liu Q., Yu J. (2026). Association of non-high-density lipoprotein cholesterol to high-density lipoprotein cholesterol ratio (NHHR) with cardiovascular mortality in peritoneal dialysis patients: A prospective cohort study. Front. Nutr..

[19] Bluemn E.G., Flahive J.M., Farber A., Bertges D.J., Goodney P.P., Eldrup-Jorgensen J., Schanzer A., Simons J.P. (2019). Analysis of Thirty-Day Readmission after Infrainguinal Bypass. Ann. Vasc. Surg..

[20] Lau B., Cole S.R., Gange S.J. (2009). Competing risk regression models for epidemiologic data. Am. J. Epidemiol..

[21] Agarwal R. (2010). Blood pressure and mortality among hemodialysis patients. Hypertension.

[22] Ekart R., Kanič V., Pečovnik Balon B., Bevc S., Hojs R. (2012). Prognostic value of 48-hour ambulatory blood pressure measurement and cardiovascular mortality in hemodialysis patients. Kidney Blood Press. Res..

[23] Kim I.S., Kim S., Yoo T.H., Kim J.K. (2023). Diagnosis and treatment of hypertension in dialysis patients: A systematic review. Clin. Hypertens..

[24] Udayaraj U.P., Steenkamp R., Caskey F.J., Rogers C., Nitsch D., Ansell D., Tomson C.R. (2009). Blood pressure and mortality risk on peritoneal dialysis. Am. J. Kidney Dis..

[25] Yılmaz Z., Yıldırım Y., Aydın F.Y., Aydın E., Kadiroğlu A.K., Yılmaz M.E., Acet H. (2014). Evaluation of fluid status related parameters in hemodialysis and peritoneal dialysis patients: Clinical usefulness of bioimpedance analysis. Medicina.

[26] Hong Y.A., Yoon H.E., Choi B.S., Shin S.J., Kim Y.S., Lee S.Y., Lee S.H., Kim S.H., Lee E.Y., Shin S.K. (2019). The Effect of Strict Volume Control Assessed by Repeated Bioimpedance Spectroscopy on Cardiac Function in Peritoneal Dialysis Patients. Sci. Rep..

[27] Park J., Campese V.M., Nobakht N., Middlekauff H.R. (2008). Differential distribution of muscle and skin sympathetic nerve activity in patients with end-stage renal disease. J. Appl. Physiol..

[28] Liu M., Takahashi H., Morita Y., Maruyama S., Mizuno M., Yuzawa Y., Watanabe M., Toriyama T., Kawahara H., Matsuo S. (2003). Non-dipping is a potent predictor of cardiovascular mortality and is associated with autonomic dysfunction in haemodialysis patients. Nephrol. Dial. Transplant..

[29] Sipahioglu M.H., Kucuk H., Unal A., Kaya M.G., Oguz F., Tokgoz B., Oymak O., Utas C. (2012). Impact of arterial stiffness on adverse cardiovascular outcomes and mortality in peritoneal dialysis patients. Perit. Dial. Int..

[30] Demirci M.S., Gungor O., Kircelli F., Carrero J.J., Tatar E., Demirci C., Kayikcioglu M., Asci G., Toz H., Ozkahya M. (2012). Impact of mean arterial pressure on progression of arterial stiffness in peritoneal dialysis patients under strict volume control strategy. Clin. Nephrol..

[31] Ramlal A.A., Naimat A., Ramakrishnan A., Pattathil M., Shahbaz T., Tariq Y., Bodige M., Irum Z., Dontul V., Ali A. (2026). Management of Hypertension in Patients Undergoing Peritoneal Dialysis: A Narrative Review. Cureus.

[32] Stergiou G., Brunström M., MacDonald T., Kyriakoulis K.G., Bursztyn M., Khan N., Bakris G., Kollias A., Menti A., Muntner P. (2022). Bedtime dosing of antihypertensive medications: Systematic review and consensus statement: International Society of Hypertension position paper endorsed by World Hypertension League and European Society of Hypertension. J. Hypertens..

[33] Smolensky M.H., Hermida R.C., Ayala D.E., Portaluppi F. (2015). Bedtime hypertension chronotherapy: Concepts and patient outcomes. Curr. Pharm. Des..

